# Why Bohmian Mechanics? One- and Two-Time Position Measurements, Bell Inequalities, Philosophy, and Physics

**DOI:** 10.3390/e20020105

**Published:** 2018-02-02

**Authors:** Nicolas Gisin

**Affiliations:** Group of Applied Physics, University of Geneva, 1211 Geneva 4, Switzerland; nicolas.gisin@unige.ch; Tel.: +41-79-776-2317

**Keywords:** Bohmian mechanics, quantum theory, surrealistic trajectories, Bell inequality

## Abstract

In Bohmian mechanics, particles follow continuous trajectories, so two-time position correlations have been well defined. However, Bohmian mechanics predicts the violation of Bell inequalities. Motivated by this fact, we investigate position measurements in Bohmian mechanics by coupling the particles to macroscopic pointers. This explains the violation of Bell inequalities despite two-time position correlations. We relate this fact to so-called surrealistic trajectories that, in our model, correspond to slowly moving pointers. Next, we emphasize that Bohmian mechanics, which does not distinguish between microscopic and macroscopic systems, implies that the quantum weirdness of quantum physics also shows up at the macro-scale. Finally, we discuss the fact that Bohmian mechanics is attractive to philosophers but not so much to physicists and argue that the Bohmian community is responsible for the latter.

## 1. Introduction

Bohmian mechanics differs deeply from standard quantum mechanics. In particular, in Bohmian mechanics, particles, here called Bohmian particles, follow continuous trajectories; hence, in Bohmian mechanics, there is a natural concept of time-correlation for particles’ positions. This led M. Correggi and G. Morchio [1] and more recently Kiukas and Werner [2] to conclude that Bohmian mechanics “cannot violate any Bell inequality” and hence is disproved by experiments. However, the Bohmian community maintains its claim that Bohmian mechanics makes the same predictions as standard quantum mechanics (at least as long as only position measurements are considered, arguing that, at the end of the day, all measurements result in position measurement, e.g., a pointer’s positions).

Here, we clarify this debate. First, we recall why two-time position correlation is at a tension with Bell inequality violation. Next, we show that this is actually not at odds with standard quantum mechanics because of certain subtleties. For this purpose, we do not go for full generality but illustrate our point with an explicit and rather simple example based on a two-particle interferometers, partly already experimentally demonstrated and certainly entirely experimentally feasible (with photons, but also feasible at the cost of additional technical complications with massive particles). The subtleties are illustrated by explicitly coupling the particles to macroscopic systems, called pointers, that measure the particles’ positions. Finally, we raise questions about Bohmian positions, about macroscopic systems, and about the large differences in appreciation of Bohmian mechanics between philosophers and physicists.

## 2. Bohmian Positions

**Figure 1:**
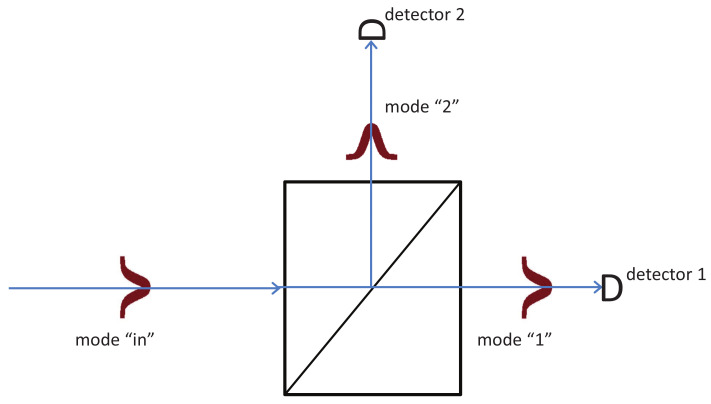
A Bohmian particle and its pilot wave arrive on a beam splitter (BS) from the left in Mode “in”. The pilot wave emerges both in Modes 1 and 2, as per the quantum state in standard quantum theory. However, the Bohmian particle emerges either in Mode 1 or in Mode 2, depending on its precise initial position. As Bohmian trajectories cannot cross each other (in configuration space), if the initial position is in the lower half of Mode “in”, then the Bohmian particle has the BS in Mode 1 or, if not, in Mode 2.

Bohmian particles have, at all times, well defined positions in our three-dimensional space. However, for the purpose of my analysis, I need only to specify in which mode the Bohmian particle is. Here I use “mode” as is usually done in optics, including atomic optics. For example, if a particle in Mode 1 encounters a beam splitter (BS) with Output Modes 1 and 2, then the Bohmian particle exits the beam splitter either in Mode 1 or in Mode 2, see Figure 1.

Part of the attraction of Bohmian mechanics lies then in the following assumption:

- Assumption **H**:
  *Position measurements merely reveal in which (spatially separated and non-overlapping) mode the Bohmian particle actually is*.

Accordingly, if Modes 1 and 2 after the beam splitter are connected to two single-particle detectors, then, if the Bohmian particle is in Mode 1, the corresponding detector clicks, and the case of Mode 2 is similar, see Figure 1.

## 3. Two-Time Position Correlation in a Bell Test

**Figure 2:**
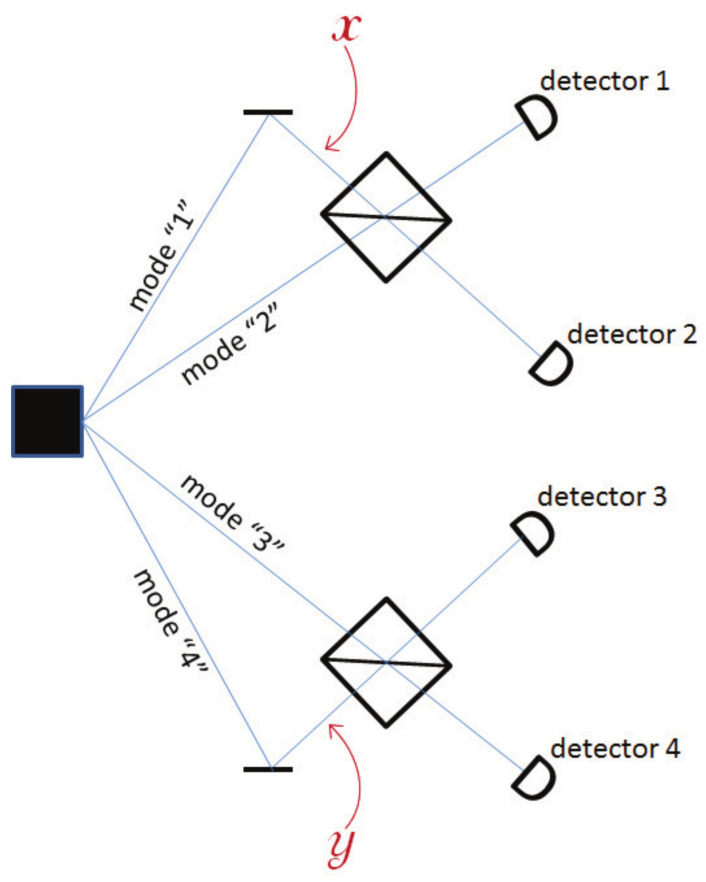
Two Bohmian particles spread over four modes. The quantum state is entangled, see Equation (1), so the two particle are either in Modes 1 and 4 or in Modes 2 and 3. Alice applies a phase *x* on Mode 1 and Bob a phase *y* on Mode 4. Accordingly, after the two beam splitters, the correlations between the detectors allow Alice and Bob to violate Bell inequality. The convention regarding mode numbering is that modes do not cross, i.e., the *n*th mode before the beam splitter goes to detector *n*.

Let’s consider a two-particle experiment with 4 modes, labeled 1, 2, 3, and 4, as illustrated in Figure 2. The source produces the quantum state:

(1) $$\psi_{0}=\left(|1001\rangle +|0110\rangle \right)/\sqrt{2}$$

where, e.g., |1001〉 means that there is one particle in Mode 1 and one in Mode 4, and Modes 2 and 3 are empty. This is an entangled state that can be used in a Bell inequality test. For this, Alice (who controls Modes 1 and 2) and Bob (who controls Modes 3 and 4) apply phases *x* and *y* to Modes 1 and 4, respectively, and combine their modes on a beam splitter, see Figure 2. Taking into account that a reflection on a BS induces a phase $e^{i\pi /2}=i$, the quantum state after the two beam splitters reads

(2) $$\begin{array}{c} \frac{e^{i(x+y)}}{2^{3/2}}\left(|1001\rangle +i|0101\rangle +i|1010\rangle -|0110\rangle \right)\\ +\;\frac{1}{2^{3/2}}\left(|0110\rangle +i|0101\rangle +i|1010\rangle -|1001\rangle \right). \end{array}$$

If Modes 1, 2, 3, and 4, after the beam splitter, encounter four single-particle detectors, also labeled 1, 2, 3, and 4, then the probabilities for coincidence detection are

(3) $$\begin{array}{c} P_{14}=P_{23}=\frac{1}{8}{\left|e^{i(x+y)}-1\right|}^{2}=\frac{1-\cos(x+y)}{4} \end{array}$$

(4) $$\begin{array}{c} P_{13}=P_{24}=\frac{1}{8}{\left|e^{i(x+y)}+1\right|}^{2}=\frac{1+\cos(x+y)}{4} \end{array}$$

from which a maximal violation of the CHSH-Bell inequality of $2\sqrt{2}$ can be obtained with appropriate choices of the phase inputs.

In Bohmian mechanics, this experiment is easily described. Denote the two particles’ positions $r_{A}$ and $r_{B}$. In the initial state (Equation (1)), the particles are either in Modes 1 and 4, a situation we denote $r_{A}\in \;$“1” and $r_{B}\in \;$“4,” or in Modes 2 and 3, i.e., $r_{A}\in \;$“2” and $r_{B}\in \;$“3.” According to Bohmian mechanics, the particles have more precise positions, but for our argument this suffices.

**Figure 3:**
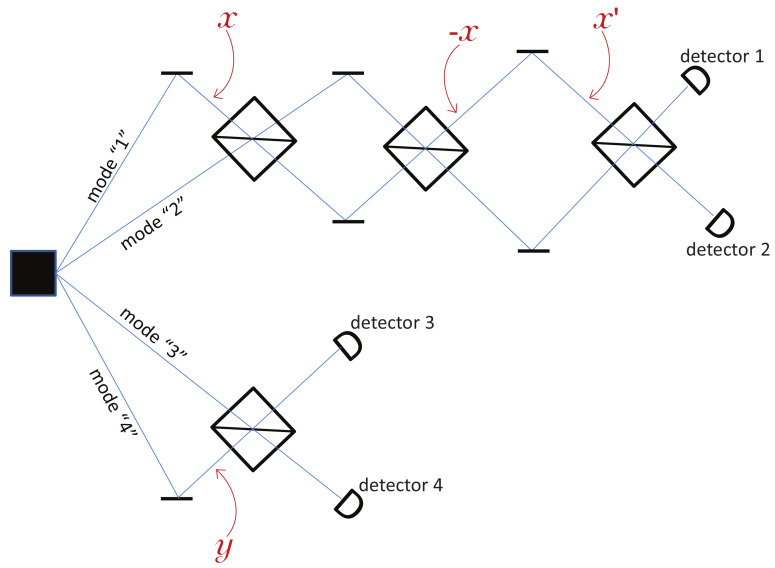
Alice’s first “measurement”, with phase *x*, can be undone because in Bohmian mechanics there is no collapse of the wavefunction. Hence, after having applied the phase −x after her second beam splitter, Alice can perform a second “measurement” with phase *x*′. Mode number convention implies, e.g., that Mode 1 is always the upper mode, i.e., the mode on which all phases *x*, −x and *x*′, are applied.

Now, according to Bohmian mechanics and Assumption **H**, one does not need to actually measure the positions of the particles; it suffices to know that each is in one specific mode. Hence, one can undo Alice’s measurement as illustrated in Figure 3. After the phase shift −x, the quantum state is precisely back to the initial state $\psi_{0}$, see Equation (1). Alice can thus perform a second measurement with a freshly chosen phase *x*′ and a third beam splitter, see Figure 3. Moreover, as Bohmian trajectories cannot cross each other (in configuration space), if $r_{A}$ is in Mode 1 before the first BS, then $r_{A}$ is also in Mode 1 before the last BS.

There is no doubt that, according to Bohmian mechanics, there is a well-defined joint probability distribution for Alice’s particle at two times and Bob’s particle: $P(r_{A},r$′${}_{A},r_{B}|x,x$′,y), where $r_{A}$ denotes Alice’s particle after the first beam splitter and *r*′${}_{A}$ after the third beam splitter of Figure 3. But here comes the puzzle. According to Assumption **H**, if $r_{A}\in \;$“1”, then any position measurement performed by Alice between the first and second beam splitter necessarily results in a=1. Similarly, $r_{A}\in \;$“2” implies a=2. Thus, Alice’s position measurement after the third beam splitter is determined by *r*′${}_{A}$, and Bob’s measurement is determined by $r_{B}$. Hence, it seems that one obtains a joint probability distribution for Alice’s measurements results and Bob’s: P(a,a′,b|x,x′,y). However, such a joint probability distribution implies that Alice does not have to make any choice (she merely makes both choices, one after the other), and in such a situation there cannot be any Bell inequality violation. Hence, as claimed in [2], it seems that the existence of two-time position correlations in Bohmian mechanics prevents the possibility of a CHSH-Bell inequality violation, in contradiction with quantum theory predictions and experimental demonstrations [3].

Let’s have a closer look at the probability distribution that lies at the bottom of our puzzle: $P(r_{A},r$′${}_{A},r_{B}|x,x$′,y). More precisely, it suffices to consider in which modes the Bohmian particles are. That is, it suffices to consider the following joint probability distribution:

(5) $$P(r_{A}\in “a”,r\prime {}_{A}\in “a^{\prime }”,r_{B}\in “b”|x,x\prime ,y)$$

where a,a′=1,2 and b=3,4 number modes. This can be computed explicitly:

(6) $$\begin{array}{c} P\left(r_{A}\in “a”,r\prime {}_{A}\in “a^{\prime }”,r_{B}\in “b”|x,x\prime ,y\right)=\frac{1+{\left(-1\right)}^{a+b}\cos\left(x+y\right)}{4}\cdot \frac{1+{\left(-1\right)}^{a\prime +b}\cos\left(x+y\right)}{2}. \end{array}$$

Note that, if one sums over *a*′, i.e., traces out Alice’s second measurement, then one recovers the quantum prediction equations (Equations (3) and (4)):

(7) $$\begin{array}{c} P(r_{A}\in “a”,r_{B}\in “b”|x,y)=\\ \sum_{a\prime }P\left(r_{A}\in “a”,r\prime_{A}\in “a^{\prime }”,r_{B}\in “b”|x,x\prime ,y\right)=\\ \frac{1+{\left(-1\right)}^{a+b}\cos\left(x+y\right)}{4}. \end{array}$$

It is important is to notice that $P(r_{A}\in “a”,r_{B}\in “b”|x,y)$ does not depend on Alice’s second measurement setting *x*′, as one would expect. Similarly, if one traces out Alice’s first measurement,

(8) $$\begin{array}{c} P\left(r_{A}\in “a^{\prime }”,r_{B}\in “b”|x\prime ,y\right)=\frac{1+{\left(-1\right)}^{a\prime +b}\cos\left(x\prime +y\right)}{4} \end{array}$$

one recovers Equations (3) and (4). Again, the probability that Equation (8) does not depend on Alice’s first measurement setting.

So far so good, but now comes the catch. If one traces out Bob’s measurement, one obtains a probability distribution for Alice’s particle’s position that depends on Bob’s setting *y*:

(9) $$\begin{array}{c} P(r_{A}\in “a”,r_{A}\in “a^{\prime }”|x,x\prime ,y)=\\ \sum_{b}P\left(r_{A}\in “a”,r\prime_{A}\in “a^{\prime }”,r_{B}\in “b”|x,x\prime ,y\right)=\\ \frac{1+{\left(-1\right)}^{a+a\prime }\cos\left(x+y\right)\cos\left(x\prime +y\right)}{4}. \end{array}$$

Hence, the joint probability distribution (Equation (6)) is signaling from Bob to Alice! Is this a problem for Bohmian mechanics? Probably not, as the Bohmian particles’ positions are assumed to be “hidden”. Actually, it is already well-known that they have to be hidden in order to avoid signaling in Bohmian mechanics. Some may find this feature unpleasant, as it implies that Bohmian particles are postulated to exist “only” to immediately add that they are ultimately not fully accessible, but this is not new.

Consequently, defining a joint probability for the measurement outcomes *a*, *a*′, and *b* in the natural way,

(10) $$\begin{array}{cc} P & (a,a\prime ,b|x,x\prime ,y)\equiv \\ P & \left(r_{A}\in “a”,r_{A}\in “a^{\prime }”,r_{B}\in “b”|x,x\prime ,y\right) \end{array}$$

can be done mathematically but cannot have a physical meaning, as P(a,a′,b|x,x′,y) would be signaling.

## 4. What Is Going on? Let’s Add a Position Measurement

In summary, it is the identification in Equation (10) that confused the authors of [1,2] and led them to wrongly conclude that Bohmian mechanics cannot predict violations of Bell inequalities in experiments involving only position measurements. Note that the identification of Equation (10) follows from Assumption **H**, so Assumption **H** is wrong. Every introduction to Bohmian mechanics should emphasize this. Indeed, Assumption **H** is very intuitive and appealing, but wrong and confusing.

**Figure 4:**
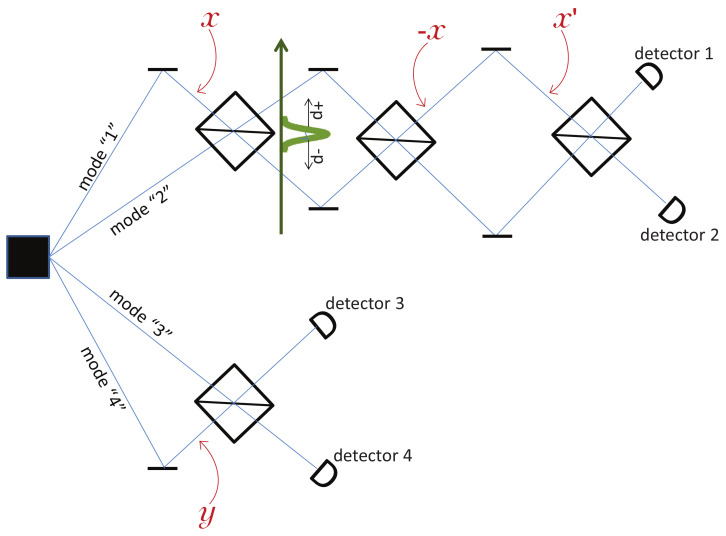
We add a pointer that measures through which path Alice’s particle propagates between her first and second beam splitter. The pointer moves up if Alice’s particle goes through the upper path, i.e., $r_{A}\in \;$“1”, and down if it goes through the lower path, i.e., $r_{A}\in \;$“2”. Hence, by finding out the pointer’s position, one learns through which path Alice’s particle goes, i.e., one finds out Alice’s first measurement result, though it all depends how fast the pointer moves. See text for explanation.

To elaborate on this, let’s add an explicit position measurement after the first beam splitter on the Alice side. The fact is that, according to both standard quantum theory and Bohmian mechanics, this position measurement perturbs the quantum state (hence the pilot wave) in such a way that the second measurement, labeled *x*′ on Figure 4, no longer shares the correlation (Equation (9)) with the first measurement, see [4,5,6].

Let’s model Alice’s first position measurement, labeled *x* (i.e., corresponding to the input phase *x*), by an extra system, called here the pointer, initially at rest in a Gaussian state, see Figure 4. One should think of the pointer as a large and massive system; note that it suffices to consider the state of the center of mass of the pointer. If Alice’s particle passes through the upper part of the interferometer ($r_{A}\in \;$“1”), then the pointer gets a kick in the upward direction and is left with a momentum +p; however, if Alice’s particle passes through the lower part of her interferometer ($r_{A}\in \;$“2”), then the pointer gets a kick −p. We made *p* large enough so that the two quantum states of the pointer |±p〉 are orthogonal, i.e., according to quantum theory, we consider a strong (projective) which-path measurement. Note, however, that, immediately after the pointer has interacted with Alice’s particle, the two Gaussians corresponding to |±p〉 overlap in space, so no position measurement can distinguish them. It is only after some time that the two Gaussians separate in space and that position measurements can distinguish them. Since in Bohmian mechanics there are only position measurements, this implies that, in Bohmian mechanics, it takes some time for the pointer to measure Alice’s particle.

Accordingly, if *p* is large enough for the pointer to have moved by more than its spread by the time Alice’s particle hits the second BS, then the pointer acts like a standard measurement, and the second position measurement *x*′ of Alice’s particle is perturbed by measurement *x*, as discussed in the previous paragraph. However, if *p* is small enough, then, by the time the second measurement *x*′ takes place, the pointer barely moves. In this case, the second position measurement is not affected [4,5,6], see also the appendix. However, it is now this second measurement *x*′ that perturbs the “first” one, i.e., perturbs measurement *x*. Indeed, because of the entanglement between Alice’s particle and the pointer, if one waits long enough for the pointer to move by more than its spread and then reads the result of the “first” measurement out of this pointer, then one will not find the expected result: the second measurement perturbed the “first” one. I put “first” in quotes because, in such a slow measurement, the result is actually read out of the pointer after the “second” measurement took place.

This is very similar to the so-called surrealistic trajectories, see [4,5,6]. In the appendix, I recall this counter-intuitive aspect of Bohmian mechanics.

## 5. What about Large Systems?

So far so good. But let’s now consider, not single particles, but elephants. One of the advantages of Bohmian mechanics is that whether systems are microscopic and macroscopic makes no difference: all systems are treated alike. The price to pay, as we illustrate below, is that all the strangeness of quantum physics at the microscopic level has to show up also at the macroscopic level.

Let’s consider two elephants in the state of Equation (1) corresponding to entangled elephants in Modes 1 & 4 superposed with elephants in Modes 2 & 3. Note that, instead of elephants, one may consider classical light pulses and replace, in Equation (1), the one-photon state |1〉 with a coherent state |α〉 with mean photon number ${\left|\alpha \right|}^{2}$ as large as desired: $\left(|\alpha ,0,0,\alpha \rangle +|0,\alpha ,\alpha ,0\rangle \right)/\sqrt{2}$. The beam splitters have to be replaced by EBSs—Elephant Beam Splitters—which split elephants: an incoming elephant emerges from an EBS in a superposition of elephant-transmitted and elephant-reflected. In the case of coherent states, the transformation reads:

(11) $$\begin{array}{c} |\alpha ,0\rangle \to \left(|\alpha ,0\rangle +i|0,\alpha \rangle \right)/\sqrt{2} \end{array}$$

(12) $$\begin{array}{c} |0,\alpha \rangle \to \left(i|\alpha ,0\rangle +|0,\alpha \rangle \right)/\sqrt{2}. \end{array}$$

Note that the above deeply differs from the standard BS, which corresponds to $|\alpha ,0\rangle \to |\alpha /\sqrt{2},i\alpha /\sqrt{2}\rangle$.

The story of the single particles described above remains the same. In Bohmian mechanics, the elephants’ positions are also hidden, or at least not fully accessible. However, this is puzzling, as it means that, when one “looks slowly” (as the pointer in Section 4, see also the Appendix) at an elephant, one may see it where it is not. Indeed, according to Bohmian mechanics, an elephant is where all the Bohmian positions of all the particles that make up the elephant are, but what does this mean if it does not correspond to where one sees the elephant? Bohmians may reply that one does not “look slowly” at elephants and that EBSs do not exist. This is certainly true of today’s technology, but there will soon be beam splitters for quantum systems large enough to be seen by the naked eye. In addition, to avoid signaling, it has to be impossible to “see” or find out in any way two-time position correlations of such quantum systems, even when they are large.

Admittedly, it is an advantage that, in Bohmian mechanics, the difference between micro- and macro-worlds is immaterial. But, accordingly and unavoidably, quantum weirdness shows up at the macro-scale.

## 6. Assumption **H** Revisited

Assumption **H** is wrong. How should one reformulate it? Clearly, a position measurement does not *merely* reveal the Bohmian particle because of the following:

1. A position measurement necessarily involves the coupling to a large system, some sort of pointer, and this coupling implies some perturbation. Hence the “merely” in assumption **H** is wrong [7].
2. Whether a position measurement reveals information about the Bohmian particle or not depends on how the coupling to a large system is done and on how that large system (the pointer) evolves. Hence, not all measurements that, according to quantum theory, are position measurements, are also Bohmian-position measurements: some quantum-position measurements do not reveal where the Bohmian particle is.

The first point above is very familiar to quantum physicists. However, it may take away some of the appeal of Bohmian mechanics. Indeed, the naive picture of particles with always well-defined positions is obscured by the fact that these positions cannot be “seen”—in fact, one can not “merely see” in which mode a Bohmian particle is. At the end of the day, Bohmian mechanics is not simpler than quantum theory. The promise of a continuously well-defined position and the associated intuition is deceptive.

The second point listed above is interesting: One should distinguish between *quantum-position* and *Bohmian-position* measurements. The latter refers to measurements that provide information about the position of Bohmian particles. It would be interesting to figure out how to characterize such Bohmian-position measurements without the need to fully compute all the Bohmian trajectories.

## 7. Why Bohmian Mechanics

From all we have seen so far, one should, first of all, recognize that Bohmian mechanics is deeply consistent and provides a nice and explicit existence proof of a deterministic non-local hidden variables model. Moreover, the ontology of Bohmian mechanics is pretty straightforward: the set of Bohmian positions is the real stuff. This is especially attractive to philosophers. Understandably so. But what about physicists mostly interested in research? What new physics did Bohmian mechanics teach us in the last 60 years? Here, I believe it is fair to answer: Not enough! Understandably disappointing.

It is deeply disappointing that an alternative theory to quantum mechanics, a theory that John Bell thought should be taught in parallel to standard textbook quantum mechanics [8], did not produce new physics, nor even inspirations for new ideas to be tested in the lab (though see [9,10,11,12]). How could this be? Some may conclude that not enough people worked on Bohmian mechanics. But tens or hundreds of passionate researchers worked on it for decades. Some may conclude that this lack of new ideas proves that Bohmian mechanics is a dead end. But how could a consistent theory, empirically equivalent to quantum theory, have no future?

Let me suggest some possible, albeit only partial, answers to the above puzzle. I am afraid that almost all the research on Bohmian mechanics over the last several decades remained trapped within an exceedingly narrow viewpoint and worked only on problems of interest that were highly specific to their Bohmian community. I believe this is especially disappointing, as there were several interesting open problems that Bohmian-inspired ideas could have addressed. The positive side is there are likely still interesting open problems that open-minded researchers can explore.

Let me illustrate some of the ideas I believe Bohmian mechanics should have triggered. This list is obviously subjective—it is only important that it is not empty. Bohmian mechanics, like quantum theory, is in deep tension with relativity theory. I know of Bohmians who claim that it is obvious that any non-local theory, Bohmian or not, requires a privileged universal reference frame. I also know of Bohmians who claim that it is obvious that Bohmian mechanics can be generalized to a relativistic theory (though, admittedly, I never understood their model). However, I know of no Bohmians who are inspired by their theory and its tension with relativity to try to go beyond Bohmian mechanics, as illustrated in the next two paragraphs.

According to Bohmian mechanics, particles “make decisions” at beam splitters in the sense that, after a beam splitter, the particle is definitively in one of the output modes. Admittedly, this is not a real decision as everything is determined by the initial state of the particle and of all other systems entangled with the particle. However, let me continue using this inspiring terminology. Accordingly, and following Suarez, we call such beam splitters *choice-devices* [13]. Such choice-devices take into account everything in their past. Now, a natural assumption inspired by the sketched description is that the past is not merely the past light cone, but all of the past in the inertial reference frame of the choice device. This idea led Suarze and Scarani to suggest that one should test situations in which several choice-devices, e.g., several beam splitters, are in relative motion such that what is the past for one choice-device may differ from the past of another choice-device [14]. This has the advantage (at least for researchers in physics) that it leads to experimental predictions that differ from standard quantum predictions and that can be experimentally tested. Hence, this brings Bohmian-inspired ideas to physics. This has been tested in my lab, and the result have shown that the idea, in spite of its appeal, is wrong [15].

Another Bohmian-inspired idea follows directly from an observation by Hiley and Bohm [16]: “it is quite possible that quantum nonlocal connections might be propagated, not at infinite speeds (as in standard Bohmian mechanics), but at speeds very much greater than that of light. In this case, we could expect observable deviations from the predictions of current quantum theory (e.g., by means of a kind of extension of the Aspect-type experiment).” Again, this can be experimentally tested [17,18,19,20]. The results put lower bounds on this hypothetical faster-than-light-but-finite speed influence, something like 10,000 to 100,000 times the speed of light. Aspect-type experiments between two sites can only either find that hypothetical speed or set lower bounds on it. However, recently we have been able to demonstrate that, by going to more parties, one can prove that either there is no such finite-but-superluminal speed or that one can use it for faster than light communication using only classical inputs and output (i.e., measurement settings and results) [21,22].

I am confident that Bohmian mechanics and other alternative views on quantum mechanics will inspire further ideas that will lead to experiments that might work to extend quantum theory. The real question is whether the Bohmian community will pursue such ideas.

## 8. Conclusions

Naive Bohmian mechanics that assumes Assumption **H** is wrong. Still, Bohmian mechanics is deeply consistent. Position measurements perturb the system, even in Bohmian mechanics. Hence, the existence of two-time position correlations is not in contradiction with possible violations of Bell inequalities.

Generally, position measurements sometimes reveal information about Bohmian positions, but never full information and sometimes none at all. Simple and handy criteria for determining when the Bohmian position measurements of a particle under test highly correlate with the position of the center of mass of some large pointer are still missing.

Bohmian mechanics is attractive to philosophers because it provides a clear ontology. However, it is not as attractive to researchers in physics. This is unfortunate because it could inspire brave new ideas that challenge quantum physics. 

## Figures and Tables

**Figure A1:**
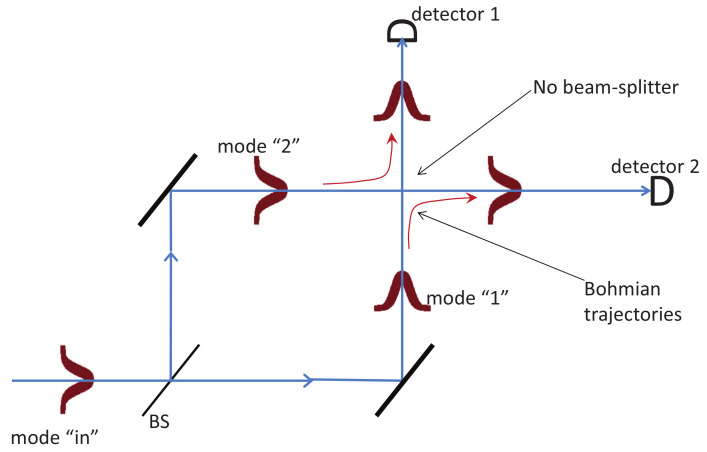
A Bohmian particle and its pilot wave arrive on a beam splitter (BS) from the left in Mode “in”. The pilot wave emerges both in Modes 1 and 2, as the quantum state in standard quantum theory. Modes 1 and 2 meet again, but there is no beam splitter at this meeting point. Nevertheless, the Bohmian trajectories bounce at this point as indicated by the red arrows. Intuitively, this can be understood because the evolution equation of the Bohmian position is a first order differential equation, so Bohmian trajectories never cross each other. This intuition is confirmed by numerical simulations.

**Figure A2:**
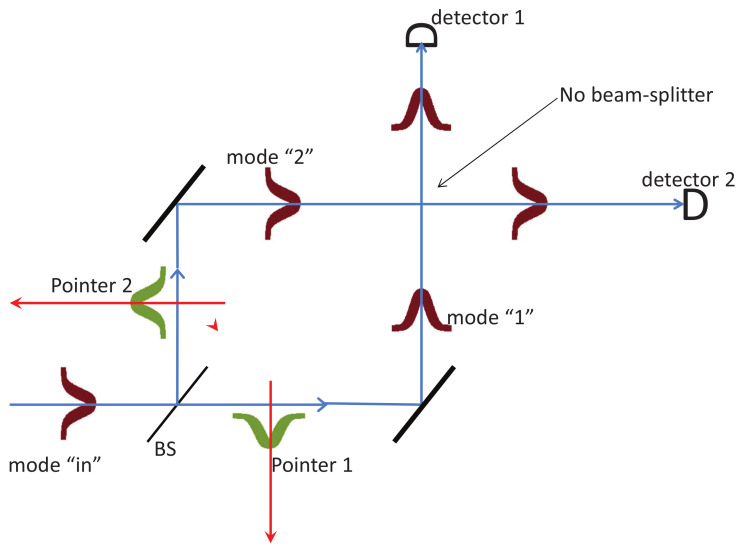
Semi-interferometer with two macroscopic pointers locally coupled to Modes 1 and 2. The pointers are initially at rest, $|p_{j}=0\rangle$, but when detecting a particle they get a kick and end in a quantum state with momentum *k*: $|p_{j}=k\rangle$.

## Appendix A. Slow Position-Measurements in Bohmian Mechanics

### Appendix A.1. Bohmian Trajectories in a Semi-Interferometer

Let us consider a “half Mach-Zehnder” interferometer, which is a Mach-Zehnder interferometer in which the second beam splitter is removed, see Figure A1. We call such a circuit a semi-interferometer. After the beam splitter, any particle entering from the input mode is in a superposition of Modes 1 and 2: $|1\rangle \to \left(|1,0\rangle +|0,1\rangle \right)/\sqrt{2}$, with possibly a relative phase irrelevant in semi-interferometers.

According to quantum theory, if Detector 1 clicks, then the particle went through Mode 1. This should be interpreted as “if one adds position measurements in Modes 1 and 2, then there is a 100% correlation between Detector 1 and the position measurement in Mode 1 (and similarly for Detector 2 and the position measurement in Mode 2)”.

According to Bohmian mechanics, things are different. If Detector 1 clicks, then the particle went through Mode 2, in sharp contrast to the quantum retro-diction. However, the interpretation is also totally different. According to Bohmian mechanics, particles follow continuous trajectories and the interpretation here is that, if Detector 1 clicks, then the Bohmian particle followed Mode 2.

In order to reconcile both views, let’s add position measurements in Modes 1 and 2.

### Appendix A.2. Position Measurements in Modes 1 and 2

In order to describe position measurements in both modes, we add two pointers, each initially at rest, denoted $|p_{j}=0\rangle$, that we locally couple to Modes 1 and 2 in such a way that, if the particle is in Mode *j*, then the corresponding pointer gets a momentum kick *k*, resulting in state $|p_{j}=k\rangle$, while the other pointer is left unaffected, see Figure A2. The joint particle-pointers state after the two local interactions thus read $\left(|1,0\rangle |p_{1}=k\rangle |p_{2}=0\rangle +|0,1\rangle |p_{1}=0\rangle |p_{2}=k\rangle \right)/\sqrt{2}$. Note that the pointers are in some localized (e.g., Gaussian) states, the kets only indicate the mean momenta.

Let us emphasize that the pointer should be thought of as large and massive and consisting of many internal degrees of freedom; in short, it is a “macroscopic” object, and the result of the position measurement can merely be read of the pointer’s position: if the pointer moves, then it has detected the presence of the particle; if the pointer hasn’t moved, then the particle went the other way. Such a formalization of position measurements applies both to quantum and Bohmian theories.

Note that, in order for the pointer to indicate an unambiguous result, one has to wait long enough for the pointer to have moved by much more than it’s spread Δx and the kick has to be large enough, k>>ℏ/Δx.

According to quantum theory, if Detector 1 clicks, then Pointer 1 got a kick and thus moves, while Pointer 2 rests in state |p=0〉. However, the situation as described by Bohmian mehcanics is more interesting.

First, consider the case that the kick *k* is so large that the pointer, if kicked, moves by more than its spread before the two Modes 1 and 2 cross at the place of the “missing beam splitter”. In this case, the particle and the kicked pointer become entangled, and this modifies the Bohmian trajectory of the particle. According to this modified trajectory and in full agreement with quantum predictions, if Detector 1 clicks, then Bohmian mechanics predicts that it is Pointer 1 that moved (including the Bohmian position of pointer 1). Note that, in this situation, Bohmian trajectories can apparently cross each other, because the trajectory actually happens in a higher dimensional (configuration) space and it is only its shadow in our space that crosses.

Next, consider the case that the kick *k* is not that large and that, by the time Modes 1 and 2 cross, the pointer has barely moved. Bohmian mechanics predicts that, if Detector 1 clicks, the particle went through Mode 2 (as if there were no pointer); however, if one waits long enough for the pointer to eventually move by more than its spread, then one finds that it is Pointer 1 that moves. Accordingly, in the case of “slow pointers”, the pointer indicates where the Bohmian particle was not. This is surprising, at least to physicists that are used to quantum theory. However, this is how Bohmian mechanics describes the situation, and one should add that there is nothing wrong with this description in the sense that all observable predictions are in agreement with quantum predictions [23,24].

Finally, we investigated numerically intermediate cases in which, at the time Modes 1 and 2 cross, the pointer moved but not much. We find that, in such cases, some trajectories of the particle bounce in the region of mode crossing, while other trajectories go through the crossing region more or less in straight lines. Accordingly, conditioned on Detector 1 clicking, there is a chance that the particle went through Mode 1 and a complementary chance that it went through Mode 2, depending on the precise value of the kick *k* and the exact initial position of the Bohmian particle.

Note that one of the two detectors moves fast to prevent the Bohmian trajectories from bouncing in the crossing region. In fact, there is only one detector; if the kick is received by this single detector, the Bohmian trajectories bounce.

Finally, note that, for pointers composed of many internal degrees of freedom, one of the pointer’s particle motion depends on whether the test particle is present or not for the counter-intuitive surreal Bohmian trajectories to disappear. However, if only the center of mass is coupled to the test particle, then there is no reason for any of the particles making up the pointer to move differently, regardless of whether or not the test particle is present.

### Appendix A.3. Conclusions

What is a position measurement? Quantum theory has a clear answer to this question. However, in Bohmian mechanics, there are two possible definitions. First, the natural one: a *Bohmian position* measurement is any interaction between the particle under test and a macroscopic device (e.g., a pointer) that fully correlates the Bohmian position of the particle immediately before the interaction took place with the final state of the device (e.g., the final position of the pointer). Next, a quantum-inspired one: Anything that is a position measurement according to quantum theory (i.e., represented by the position operator *q* or a function of it) is also a *quantum-position* measurement in Bohmian mechanics.

Hence, in Bohmian mechanics, one should distinguish between Bohmian-position measurements and quantum-position measurements. In most situations, both types of position measurements coincide. However, there are cases, such as the slow pointer described in this note, where the two starkly differ.

When one says that Bohmian mechanics makes the same predictions as quantum theory as long as all measurements, at the end of the day, reduce to position measurements, one refers to quantum-position measurements. This may differ from Bohmian-position measurements, so Bohmian trajectories may differ from quantum expectations. This is surprising to quantum physicists, but one should emphasize that there is nothing wrong with that: different theories lead to different pictures of reality.

## References

[1] Correggi M., Morchio G. (2002). Quantum mechanics and stochastic mechanics for compatible observables at different times. Ann. Phys..

[2] Kiukas J., Werner R.F. (2010). Maximal violation of Bell inequalities by position measurements. arXiv.

[3] Brunner N., Cavalcanti D., Pironio S., Scarani V., Wehner S. (2014). Bell nonlocality. Rev. Mod. Phys..

[4] Englert B.-G., Scully M.O., Süssmann G., Walther H. (1992). Surrealistic Bohm Trajectories. Z. Naturforschung A.

[5] Vaidman L. (2005). The reality in Bohmian quantum mechanics or can you kill with an empty wave bullet?. Found. Phys..

[6] Dewdney C., Hardy L., Squires E.J. (1993). How late measurements of quantum trajectories can fool a detector. Phys. Lett. A.

[7] Maudlin T. (2016). Personal communication.

[8] Bell J.S. (1982). On the impossible pilot wave. Found. Phys..

[9] Valentini A. (2005). Hidden Variables and the Large-Scale Structure of Spacetime. arXiv.

[10] Mahler D.H., Rozema L., Fisher K., Vermeyden L., Resch K.J., Wiseman H.M., Steinberg A. (2016). Experimental nonlocal and surreal Bohmian trajectories. Sci. Adv..

[11] Dürr D., Goldstein S., Zanghí N. (1992). Quantum equilibrium and the origin of absolute uncertainty. J. Stat. Phys..

[12] Goldstein S., Zanghi N., Albert D., Ney A. (2012). Reality and the Role of the Wave Function in Quantum Theory. The Wave Function: Essays in the Metaphysics of Quantum Mechanics.

[13] Suarez A. (1997). Relativistic nonlocality in an experiment with 2 *non-before* impacts. Phys. Lett. A.

[14] Suarez A., Scarani V. (1997). Does entanglement depend on the timing of the impacts at the beam splitters?. Phys. Lett. A.

[15] Stefanov A., Zbinden H., Gisin N., Suarez A. (2002). Quantum correlations with spacelike separated beam splitters in motion: Experimental test of multisimultaneity. Phys. Rev. Lett..

[16] Bohm D., Hiley B.J. (1993). The Undivided Universe.

[17] Scarani V., Tittel W., Zbinden H., Gisin N. (2000). The speed of quantum information and the preferred frame: Analysis of experimental data. Phys. Lett. A.

[18] Salart D., Baas A., Branciard C., Gisin N., Zbinden H. (2008). Testing spooky action at a distance. Nature.

[19] Cocciaro B., Faetti S., Fronzoni L. (2011). A lower bound for the velocity of quantum communications in the preferred frame. Phys. Lett. A.

[20] Yin J., Cao Y., Yong H.-L., Ren J.-G., Liang H., Liao S.-K., Zhou F., Liu C., Wu Y.-P., Pan G.-S. (2013). Lower Bound on the Speed of Nonlocal Correlations without Locality and Measurement Choice Loopholes. Phys. Rev. Lett..

[21] Bancal J.-D., Pironio S., Acin A., Liang Y.-C., Scarani V., Gisin N. (2012). Quantum non-locality based on finite-speed causal influences leads to superluminal signaling. Nat. Phys..

[22] Barnea T., Bancal J.D., Liang Y.C., Gisin N. (2013). Tripartite quantum state violating the hidden-influence constraints. Phys. Rev. A.

[23] Lam M.M., Dewdney C. (1990). Locality and nonlocality in correlated two-particle interferometry. Phys. Lett. A.

[24] Guay E., Marchildon L. (2003). Two-particle interference in standard and Bohmian quantum mechanics. J. Phys. A.

